# Mortality and Exposure Response among 14,458 Electrical Capacitor Manufacturing Workers Exposed to Polychlorinated Biphenyls (PCBs)

**DOI:** 10.1289/ehp.9175

**Published:** 2006-06-22

**Authors:** Mary M. Prince, Avima M. Ruder, Misty J. Hein, Martha A. Waters, Elizabeth A. Whelan, Nancy Nilsen, Elizabeth M. Ward, Teresa M. Schnorr, Patricia A. Laber, Karen E. Davis-King

**Affiliations:** National Institute for Occupational Safety and Health, Centers for Disease Control and Prevention, U.S. Department of Health and Human Services, Cincinnati, Ohio, USA

**Keywords:** cancer, electrical capacitor manufacturing, liver cancer, mortality, occupational exposure, PCBs, polychlorinated biphenyls, prostate cancer

## Abstract

**Background:**

We expanded an existing cohort of workers (*n* = 2,588) considered highly exposed to polychlorinated biphenyls (PCBs) at two capacitor manufacturing plants to include all workers with at least 90 days of potential PCB exposure during 1939–1977 (*n* = 14,458). Causes of death of *a priori* interest included liver and rectal cancers, previously reported for the original cohort, and non-Hodgkin lymphoma (NHL), melanoma, and breast, brain, intestine, stomach, and prostate cancers, based on other studies.

**Methods:**

We ascertained vital status of the workers through 1998, and cumulative PCB exposure was estimated using a new job exposure matrix. Analyses employed standardized mortality ratios (SMRs; U.S., state, and county referents) and Poisson regression modeling.

**Results:**

Mortality from NHL, melanoma, and rectal, breast, and brain cancers were neither in excess nor associated with cumulative exposure. Mortality was not elevated for liver cancer [21 deaths; SMR 0.89; 95% confidence interval (CI), 0.55–1.36], but increased with cumulative exposure (trend *p*-value = 0.071). Among men, stomach cancer mortality was elevated (24 deaths; SMR 1.53; 95% CI, 0.98–2.28) and increased with cumulative exposure (trend *p*-value = 0.039). Among women, intestinal cancer mortality was elevated (67 deaths; SMR 1.31; 95% CI, 1.02–1.66), especially in higher cumulative exposure categories, but without a clear trend. Prostate cancer mortality, which was not elevated (34 deaths; SMR 1.04; 95% CI, 0.72–1.45), increased with cumulative exposure (trend *p*-value = 0.0001).

**Conclusions:**

This study corroborates previous studies showing increased liver cancer mortality, but we cannot clearly associate rectal, stomach, and intestinal cancers with PCB exposure. This is the first PCB cohort showing a strong exposure–response relationship for prostate cancer mortality.

U.S. production and use of polychlorinated biphenyls (PCBs) ended in 1977 (Smith and Brown 1987). Concern continues about the persistence of PCBs in the environment and potential human health risks. Several strains of rats exposed to PCBs had preneoplastic changes of the biliary tract, intestine, and stomach (Morgan et al. 1981; National Cancer Institute 1978) and increased incidence of liver tumors (Carpenter 2000; Kimbrough et al. 1975). Liver toxicity and hepatocellular neoplasm incidence differed between PCB mixtures, were more severe in females than in males, and increased with dose in females (Mayes et al. 1998).

The International Agency for Research on Cancer (IARC 1987) concluded that there is sufficient evidence of carcinogenicity of PCBs in animals but limited evidence in humans. Studies of PCB-exposed workers have generally found excesses for several cancer sites, but many were limited by sample size, imprecision in measuring PCB exposure, and/or inability to control for other risk factors and confounders (Carpenter 2000; Faroon et al. 2001).

The mortality of 2,588 workers considered highly exposed to PCBs at two electrical capacitor plants in New York (plant 1) and Massachusetts (plant 2) was initially studied through 1975 (Brown and Jones 1981) and later updated through 1982 (Brown 1987) and 1998 (Prince et al. 2006). In the current study we expanded this cohort to include all employees who worked ≥ 90 days, and we ascertained their vital status through 1998. We used a semiquantitative job-exposure matrix (JEM) to estimate cumulative PCB exposure.

Our goal was to investigate previously reported mortality excesses for cancers of the biliary passages, liver, and gallbladder (henceforth, liver cancer) and rectum (Brown 1987). Other *a priori* outcomes of interest included all-cancer mortality (Bertazzi et al. 1987), non-Hodgkin lymphoma (NHL) (Rothman et al. 1997; Hardell et al. 2001), breast cancer (Falck et al. 1992), melanoma and brain cancer (Loomis et al. 1997; Sinks et al. 1992), prostate cancer (Charles et al. 2003; Ritchie et al. 2003) and stomach and intestinal cancer (Mallin et al. 2004).

## Methods

The expanded cohort included all workers with ≥ 90 days of employment at plant 1 between 1 January 1946 and 30 June 1977, or at plant 2 between 1 January 1939 and 29 March 1976. For plant 1, work histories indicated whether jobs were salaried or hourly positions; those for plant 2 did not. Based on an examination of job titles, all plant 2 workers were considered hourly. Salaried workers at plant 1 were included in the expanded cohort because many had both hourly and salaried experience and may have worked alongside hourly employees. In analyses restricted to plant 1 hourly workers, we classified employees as “hourly” if they had been employed ≥ 90 days in nonsalaried jobs. Employment records provided Social Security numbers, demographic information, and work histories. For plant 1, we used Internal Revenue Service Employer’s Quarterly Earning Reports (form 941) and pension rosters to verify completeness of ascertainment. Plant 1 cohort members included workers studied by Kimbrough et al. (1999, 2003).

Demographic information (race, sex, birth date) was corrected or updated based on data from 4,300 interviews for a cohort breast cancer incidence study. Race was routinely verified and/or updated for deceased workers based on death certificates. Employment records did not include race information. Many plant 2 workers were possibly of mixed ethnic origin (Cape Verdean). Workers with only employment records were assigned “unknown” race, whereas workers for whom we had death certificates or interview data were assigned distinct race codes of white, black, Asian, Hispanic, or American Indian. Workers coded as black, Asian, or American Indian were assumed to be nonwhite; based on area demographics, all other workers were assumed to be white.

This study added 11,870 workers to the original cohort of 2,588 (Prince et al. 2006), and ascertained vital status through 31 December 1998, by linking to the Social Security Administration, the Internal Revenue Service, the National Death Index (NDI; National Center for Health Statistics 1999), the U.S. Postal Service, and credit bureaus. Causes of death were obtained from NDI Plus or coded by a qualified nosologist from death certificates according to the *International Classification of Diseases* (ICD) revision in effect at the time of death [data are presented according to the ninth revision (ICD-9; World Health Organization 1977)]. Because the NDI excludes deaths before 1979, workers lost to follow-up before 1979 were classified as “vital status unknown” and considered alive until the date last observed. Workers not lost to follow-up before 1979 and not reported as deceased were considered alive until the study end date. This study was approved by the National Institute for Occupational Safety and Health (NIOSH) Human Subjects Review Board.

Commercial PCB mixes, 41–54% chlorine (Brown and Jones 1981), were used at both plants, with less chlorinated varieties used more recently. At any given time, more than one mix likely was in use. NIOSH conducted exposure surveys in spring 1977. Time-weighted average air samples ranged from 24–476 μg/m^3^ at plant 1 and 50–1,260 μg/m^3^ at plant 2 (Brown and Jones 1981).

We developed plant-specific semiquantitative JEMs using job descriptions, production factors, process information, PCB usage eras, industrial hygiene air sampling data, and exposure determinants such as proximity to bulk PCB sources, process temperature, frequency and duration of tasks involving contact with PCB oil, and other chemical exposures (Nilsen et al. 2004, 2005). Jobs were assessed separately for potential inhalation and dermal exposures. We used plant-specific air concentrations to assign values to the qualitative (high, medium, low, background) inhalation and dermal exposure ratings, with dermal exposure values unitless. A combined JEM averaged inhalation and dermal exposure scores because workers typically were exposed by both routes. Like its dermal component, this combined metric is unitless; cumulative exposure is a number of unit-days of exposure.

We evaluated concomitant exposure to other chemicals. Trichloroethylene (TCE) has been associated with liver cancer risk (Wartenberg et al. 2000). Only workers who cleaned capacitors or performed degreasing had consistently high TCE exposures. Data suggest that TCE degreasing occurred during most years of PCB usage at plant 1 but ended by the mid-1970s. Plant 2 used TCE throughout the period of PCB exposure, but pre-1950 work histories lacked the specificity needed to identify jobs with potential TCE exposure. In the mid-1970s, TCE levels at both plants were generally below the Occupational Safety and Health Administration permissible exposure limit of 100 ppm [Agency for Toxic Substances and Disease Registry (ATSDR) 2001; Brown and Jones 1981]. Some workers at both plants also had exposures to lead and zinc during soldering; plant 1 workers had exposures to toluene and methyl butyl ketone during painting, and to aluminum and iron during welding (Brown and Jones 1981).

We used the NIOSH personal computer life table analysis system (Steenland et al. 1998) to compute person-years at risk (PYAR), expected numbers of deaths, and standardized mortality ratios (SMRs) based on U.S. and appropriate state and county rates. PYAR accumulation started on the 90th day of employment during the PCB exposure period or the date the rate files began, whichever was later; PYAR ended at death, date lost to follow-up, or the study end date (31 December 1998), whichever was earliest. In addition to analysis of underlying cause of death, all causes listed on the death certificates were analyzed using multiple cause mortality methods (Steenland et al. 1992). For myeloma, NHL, and melanoma, U.S. rate files begin on 1 January 1960; multiple cause and state and county death rate files also begin on this date. For other causes of death, U.S. rate files begin on 1 January 1940.

For tests of trend, we used quartiles of cumulative exposure among deceased workers (150, 620 and 2,300 unit-years) to stratify observed deaths and PYAR. All deaths and all cancer deaths were partitioned approximately equally into these quartiles; specific cancers (i.e., liver and prostate) were not necessarily equally partitioned. Trend tests were conducted for all *a priori* outcomes and those found to be in excess among long-term workers [≥ 10 years employment and ≥ 20 years time since first exposure (TSFE)]. Internal comparisons used multivariate Poisson regression modeling with SAS 9 software (SAS Institute 2006) to calculate rate ratios (RRs) for three higher exposure categories relative to the lowest. Poisson regression models examining cumulative exposure and mortality were adjusted for sex, age, and calendar year. Cumulative exposure was lagged for zero, 10, and 20 years. Categories of age, calendar year, or cumulative exposure were combined as necessary to eliminate categories with zero observed deaths. Workers with unknown job information (16 in plant 1 and 470 in plant 2) were excluded from exposure–response analyses.

To eliminate possible confounding due to concomitant TCE exposure, supplemental analyses for certain cancers excluded workers who ever performed degreasing. A sensitivity analysis categorized Portuguese-surnamed workers (a surrogate for Cape Verdean ancestry) as nonwhite to determine if SMRs would have changed if ethnicity were misclassified (approximately 32% of plant 2 workers and < 0.5% of plant 1 workers had Portuguese surnames).

## Results

The employment criterion was met by 14,458 workers (Table 1). Loss to follow-up rates were similar by sex across the two plants (5–6%; results not shown). At plant 1, duration of employment was similar for males and females (medians, 2.3 and 2.1 years, respectively), but cumulative PCB exposure was generally higher among males than among females (medians, 280 and 140 unit-years, respectively); however, at plant 2, males generally worked fewer years than females (medians, 1.3 and 2.0 years, respectively), but cumulative PCB exposure was similar for males and females (medians, 340 and 360 unit-years, respectively). Only 550 workers (4% overall; 7.4% in plant 1; 0.5% in plant 2) had known TCE degreasing activities (lack of specificity in pre-1950 job data may be responsible for the smaller proportion in plant 2). At both plants, 91% of the cohort had ≥ 20 years TSFE and 83% of deaths and 88% of cancer deaths occurred among workers with ≥ 20 years TSFE.

Table 2 describes the overall mortality experience of the cohort, based on U.S. mortality rates; results were similar using state and county rates, except as noted below. Total mortality was less than expected.

Overall, no *a priori* outcome of interest (i.e., all cancers; cancers of the rectum, liver, intestine, stomach, breast, prostate, and brain; melanoma; NHL) was significantly elevated in the cohort. Melanoma mortality was elevated among plant 1 workers. Stomach cancer mortality among men and intestinal cancer mortality among women were elevated, (more elevated using U.S. referents, less elevated using state and county referents. Myeloma mortality, not of *a priori* interest, was elevated primarily among men and plant 1 workers.

Chronic and unspecified bronchitis mortality was elevated, primarily among women and plant 1 workers. Mortality from “other nervous system diseases” (Parkinson disease and other neurodegenerative diseases) was elevated among women. Ischemic heart disease mortality was not reduced overall, and it was higher among plant 2 workers.

Mental disorders, other diseases of the heart (a category including cardiomyopathy and congestive heart failure), and diseases of the respiratory, digestive, and genitourinary systems had lower than expected mortality. Mortality from accidents, suicide, and homicide was quite low using U.S. referent rates but less so with state or county rates.

SMRs for “long-term” employees were elevated for total cancer in plant 2 [168 deaths; SMR, 1.13; 95% confidence interval (CI), 0.97–1.32], cancers of the rectum, liver, stomach in men, breast, female genital organs, and prostate; and chronic and unspecified bronchitis, whereas SMRs similar to those of the total cohort or reduced, were observed for cancers of the intestine and brain, melanoma, NHL, and myeloma (results not shown).

Table 3 shows results of the internal exposure–response analyses. For total cancer, RRs increased with increasing exposure across lagging categories; the trends for 0- and 10-year lags were statistically significant, and the 20-year trend was of borderline statistical significance. We observed a positive trend for liver cancer SMRs with increasing cumulative exposure. Liver cancer RRs in lagged analyses, especially the 20-year lag, were suggestive of a trend. This trend persisted in subsequent analyses (results not shown) that excluded either workers who were potentially exposed to TCE or salaried workers. We did not observe exposure–response trends across lagging categories for mortality from cancers of the stomach, intestine, rectum, breast, and brain; melanoma; NHL; and myeloma.

Exposure–response analyses suggested a positive association between stomach cancer mortality and cumulative exposure. Among men, positive trends with cumulative exposure were statistically significant in the unlagged and 10-year-lagged analyses. These trends persisted in analyses that excluded either workers who were potentially exposed to TCE or salaried workers (results not shown). Mortality from intestinal cancer was elevated relative to the referent group, particularly among women; however, trend tests for intestinal cancer among women were not statistically significant.

We observed a positive trend for prostate cancer mortality with increasing cumulative exposure in internal analyses, whether lagging for 0, 10, or 20 years. The trend was observed consistently in subsequent analyses that examined multiple causes of death or excluded either workers who were potentially exposed to TCE or salaried workers (results not shown).

Mortality from female genital organ cancers—which was elevated among long-term workers (results not shown) but has not been reported in the literature as associated with PCB exposure—was examined in internal analyses (Table 3). There were suggestions of trends in mortality with cumulative exposure for cancers of other and unspecified parts of the uterus and ovary, fallopian tube, and broad ligament but not for cervical cancer (results not shown). RRs for uterine cancer were elevated but were highly unstable. RRs for ovarian cancer were elevated, and the trend tests became more pronounced with increased lag period or exclusion of salaried workers (results not shown).

## Discussion

The relationship between cumulative exposure to PCBs and liver cancer mortality reported here corroborates the study of the most highly exposed workers in this cohort (Prince et al. 2006), as well as other studies (Bertazzi et al. 1987; Greenland et al. 1994; Mallin et al. 2004). However, we found no evidence that previously reported excesses of rectal cancer in that subcohort were related to PCB exposure (Brown 1987; Brown and Jones 1981). For cumulative PCB exposure, we also found *a*) an exposure–response relationship with overall cancer mortality; *b*) a strong exposure–response relationship with prostate cancer mortality; *c*) suggestive evidence of an association with mortality from myeloma, ovarian cancer, and stomach cancer among men; and *d*) equivocal evidence for an association with mortality from intestinal cancer among women. We found no significant mortality excesses in this cohort for NHL, melanoma, or cancers of the brain and breast, contrary to some studies (Falck et al. 1992; Loomis et al. 1997; Rothman et al. 1997; Ruder et al. 2006), but consistent with mortality studies of plant 1 of this cohort (Kimbrough et al. 1999, 2003).

Associations between environmental or occupational exposures and prostate cancer have been reported in the literature (Hessel et al. 2004; Zeegers et al. 2004), but less is known about PCB effects. In a prostate cancer case–control study, Ritchie et al. (2003) reported a weak exposure response for lipid-adjusted serum levels of PCB congener 180, whereas in a nested case–control study of U.S. electrical utility workers, Charles et al. (2003) found that prostate mortality was not associated with frequency and duration of employment in PCB-exposed jobs, and only minor elevations in risk were observed at the highest PCB exposure levels. The present study provides the strongest evidence to date for an association of prostate cancer with cumulative PCB exposure in an occupational cohort. The observed exposure–response relationship in this study has biological plausibility if the mechanism for PCB-induced prostate cancer is related to disruption of estrogenic pathways (Charles et al. 2003; Ritchie et al. 2003). Further study of the association of prostate cancer with PCB exposure will be conducted in this cohort using cancer incidence data.

Other than radiation and petrochemicals, few occupational or environmental risk factors have been definitively associated with risk of myeloma (American Cancer Society 2005). To our knowledge, this is the first occupational cohort mortality study to report an association between PCB exposure and myeloma. The analyses provide suggestive evidence that PCB exposure may have contributed to the observed excess myeloma mortality.

Studies of neurologic effects among PCB-exposed workers are limited. “Other nervous system diseases” mortality was elevated among cohort women, but without a trend with cumulative exposure (results not shown). We have analyzed specific neurodegenerative diseases separately (Steenland et al. 2006).

TCE exposure is considered a potential confounder for liver cancer mortality (Hansen et al. 2001; Motohashi et al. 1999) and possibly for stomach and prostate cancer and myeloma (Wartenberg et al. 2000). Supplemental analyses excluding workers potentially exposed to TCE were similar to those of the full cohort, suggesting that observed excesses were not related to known TCE exposure.

The present study has the typical limitations of occupational cohort mortality studies. Possible exposure misclassification cannot be ruled out because PCB exposure was assessed by job title, department, activities, and air monitoring levels rather than with measured congener-specific body burdens. The JEM provided cumulative exposure scores for relative ranking of exposure histories. To reduce possible exposure misclassification bias, we limited exposure–response analyses to the 97% of workers with known job histories.

Given our lack of information on non-occupational risk factors, it is important to consider whether the observed results could be explained by regional differences in disease burden, lifestyle, ethnicity, or potential exposure to other cancer-causing chemicals. SMRs based on U.S., state, and county rate files for liver and prostate cancer and myeloma were similar, and exposure–response relationships with cumulative exposure were robust in supplemental analyses. Therefore, findings are not likely to be due to regional differences in risk of these cancers.

Smoking, alcohol use, and diet are potential risk factors for liver, intestinal, and stomach cancers (Wingo et al. 2003) but have not been consistently associated with prostate cancer (Hickey et al. 2001; Platz et al. 2004) or myeloma (Brown et al. 1997). The effect of lifestyle factors on gastrointestinal cancers is expected to be small because cohort mortality from other conditions associated with lifestyle risks, such as heart and respiratory diseases and cirrhosis of the liver, was less than expected or as expected.

Ethnic differences in cancer risk must be considered because the ethnic composition of the plant 2 workforce reflected demographics of southeast Massachusetts, with a large population of Cape Verdean and Portuguese immigrants and their descendants (Brehm et al. 2002). Cape Verde may share with the rest of West Africa a substantial risk of liver cancer, retained by first generation emigrants (London and McGlynn 1996; Parkin et al. 2003). Because most Cape Verdeans in this cohort were U.S. born, their rates of stomach, liver, and intestinal cancers likely would be more similar to U.S. rates; there is some evidence that cancer rates in second-generation migrants resemble those in their adopted country rather than in their native country (Wingo et al. 2003).

Possible misclassification of ethnicity as a factor in the observed excess liver cancer cannot be directly evaluated because we have limited race-specific data. A sensitivity analysis used Portuguese surnames as a surrogate for Cape Verdean ethnicity, addressing whether race could have been misclassified for Cape Verdean workers. In analyses in which those with Portuguese surnames were temporarily coded as “nonwhite,” SMRs were similar to those in the current analysis (results not shown).

Given the multiple outcomes examined, significant positive associations could have occurred by chance (Shields 2006). Positive associations lacking exposure response are less likely to be causal in nature and require further corroboration.

Selection bias due to the healthy worker effect, in conjunction with low statistical power for certain causes of death, may have reduced our ability to detect increases in mortality caused by PCB exposure. However, the healthy worker effect, associated with greatly decreased heart disease mortality, is not strong in this cohort, especially in plant 2 (Table 2), and internal comparisons reduced healthy worker selection effect (Richardson et al. 2004). Because 75% of workers left the plants before the age of 55 years—presumably for other jobs rather than retirement—we cannot control for healthy worker survival by including time since last employment in our models.

The major strengths of the present study include the large number of workers, the 59 years of follow-up, and the analysis of mortality with estimated cumulative exposure. The relatively high proportion of women (> 50%) also provided a unique opportunity to examine differential mortality risk by sex (Huff 2001) in relation to cumulative PCB exposure.

The present study adds to the body of knowledge regarding PCB-related health effects and corroborates previous studies showing an increase in mortality from liver cancers; however, we cannot clearly associate intestinal and rectal cancers with PCB exposure. Melanoma mortality was moderately elevated, especially in men and plant 1 workers, but with no evidence of an exposure–response relationship.

To our knowledge, this is the first occupational cohort study showing a strong exposure–response relationship between cumulative PCB exposure and mortality from prostate cancer. It also provides suggestive evidence that excess mortality risk from myeloma, stomach cancer among men, and ovarian cancer may be associated with long-term occupational exposure to PCBs.

## Figures and Tables

**Table 1:** Characteristics of PCB-exposed workers employed ≥ 90 days.^a^

Characteristic	Plant 1	Plant 2	Total
No. of workers	6,941	7,517	14,458
Sex/race, *n* (%)		
Male/white	3,971 (57)	2,506 (33)	6,477 (45)
Male/nonwhite	4 (< 1)	16 (< 1)	20 (< 1)
Female/white	2,938 (42)	4,834 (64)	7,772 (54)
Female/nonwhite	28 (< 1)	161 (2)	189 (1)
Vital status as of 31 December 1998, *n* (%)		
Deceased, cause of death known	1,514 (22)	1,760 (23)	3,274 (23)
Deceased, cause of death unknown	72 (1)	71 (1)	143 (1)
Alive	5,021 (72)	5,269 (70)	10,290 (71)
Unknown	334 (5)	417 (6)	751 (5)
Pay status, *n* (%)		
Hourly	5,244 (76)	7,517 (100)	12,761 (88)
Salaried	1,697 (24)	0 (0)	1,697 (12)
Duration of employment,^b^*n* (%)		
Median (range), years	2.2 (0.25–31.5)	1.7 (0.25–37.0)	1.9 (0.25–37.0)
90 days to < 2 years	3,342 (48)	4,014 (53)	7,356 (51)
2–9 years	2,136 (31)	2,198 (29)	4,334 (30)
≥ 10 years	1,463 (21)	1,305 (17)	2,768 (19)
Cumulative exposure^c^ (unit-years), *n* (%)		
Unknown	16 (< 1)	470 (6)	486 (3)
< 150	2,985 (43)	2,092 (28)	5,077 (35)
150 to < 620	1,805 (26)	2,263 (30)	4,068 (28)
620 to < 2,300	1,264 (18)	1,414 (19)	2,678 (19)
≥ 2,300	871 (13)	1,278 (17)	2,149 (15)
Years since first employment, *n* (%)		
Median (range)	32.6 (0.25–53.0)	37.5 (0.25–60.0)	34.5 (0.26–60.0)
< 20 years	601 (9)	684 (9)	1,285 (9)
20–39 years	4,337 (62)	3,872 (52)	8,209 (57)
≥ 40 years	2,003 (29)	2,961 (39)	4,964 (34)
Years since last employment		
Median (range)	26.7 (0–52.7)	31.7 (0–53.6)	29.4 (0.0–53.6)
Person-years at risk	227,131	268,992	496,123

a All workers with at least 90 days of employment during the period when PCBs were in use, except those missing date of birth (*n* = 52) or date of death (*n* = 1).

b Time worked while PCBs were in use at the plants.

c Estimated using the combined inhalation–dermal job exposure matrix; 486 workers had “unknown” cumulative exposure because each had at least one job with an unknown code.

**Table 2:** Mortality among PCB-exposed workers employed ≥ 90 days, 1940–1998.^a^

	Total cohort														
	All workers			Male			Female			Plant 1			Plant 2		
Underlying cause of death^b^ (ICD-9 code)	Obs	SMR	95% CI	Obs	SMR	95% CI	Obs	SMR	95% CI	Obs	SMR	95% CI	Obs	SMR	95% CI
All deaths	3,417	0.93**	0.90–0.96	1,674	0.88**	0.84–0.93	1,743	0.97	0.93–1.02	1,586	0.87**	0.83–0.92	1,831	0.98	0.94–1.03
All cancers (140–208)	1,015	1.00	0.94–1.06	438	0.95	0.86–1.04	577	1.04	0.96–1.13	473	0.95	0.87–1.04	542	1.04	0.96–1.13
MN of buccal cavity and pharynx (140–149)	19	1.04	0.63–1.63	12	1.04	0.54–1.82	7	1.04	0.42–2.14	14	1.44	0.79–2.42	5	0.59	0.19–1.37
MN of digestive organs and peritoneum (150–159)	242	1.05	0.92–1.19	109	0.97	0.79–1.16	133	1.13	0.95–1.34	114	1.00	0.83–1.20	128	1.10	0.92–1.30
Esophagus (150)	17	0.99	0.57–1.58	12	0.98	0.51–1.71	5	1.00	0.32–2.34	6	0.63	0.23–1.37	11	1.43	0.71–2.56
Stomach (151)	31	1.13	0.77–1.60	24	1.53	0.98–2.28	7	0.59	0.24–1.22	12	0.86	0.45–1.51	19	1.40	0.84–2.19
Intestine, excluding rectum (152–153)	106	1.16	0.95–1.41	39	0.97	0.69–1.33	67	1.31*	1.02–1.66	53	1.21	0.90–1.58	53	1.12	0.84–1.47
Rectum (154)	21	1.14	0.70–1.74	8	0.86	0.37–1.69	13	1.42	0.75–2.43	11	1.20	0.60–2.15	10	1.07	0.51–1.96
Biliary passages, liver, and gallbladder (155–156)	21	0.89	0.55–1.36	7	0.63	0.25–1.30	14	1.12	0.61–1.87	9	0.78	0.36–1.48	12	0.99	0.51–1.73
Pancreas (157)	44	0.91	0.66–1.22	18	0.79	0.47–1.25	26	1.02	0.67–1.50	21	0.89	0.55–1.35	23	0.94	0.59–1.41
MN respiratory system (160–165)	272	0.95	0.84–1.07	139	0.82*	0.69–0.97	133	1.15	0.96–1.36	133	0.88	0.73–1.04	139	1.04	0.87–1.23
Trachea, bronchus, and lung (162)	256	0.93	0.82–1.05	127	0.78**	0.65–0.93	129	1.15	0.96–1.36	125	0.86	0.71–1.02	131	1.02	0.85–1.21
MN of breast (174–175)	111	0.95	0.78–1.15	0	—	—	111	0.96	0.79–1.15	39	0.91	0.65–1.24	72	0.98	0.77–1.23
MN of female genital organs (179–184)	75	1.06	0.84–1.33	NA	NA	NA	75	1.06	0.84–1.33	29	1.13	0.76–1.63	46	1.02	0.75–1.36
Cervix uteri (180)	22	1.29	0.81–1.95	NA	NA	NA	22	1.29	0.81–1.95	10	1.65	0.79–3.03	12	1.09	0.56–1.90
Other and unspecified parts of the uterus (179, 181–182)	15	0.99	0.56–1.64	NA	NA	NA	15	0.99	0.56–1.64	6	1.11	0.41–2.42	9	0.93	0.42–1.76
Ovary, fallopian tube, and broad ligament (183)	35	0.96	0.67–1.34	NA	NA	NA	35	0.96	0.67–1.34	11	0.82	0.41–1.47	24	1.05	0.67–1.56
MN of prostate (185)	34	1.04	0.72–1.45	34	1.04	0.72–1.45	NA	NA	NA	21	1.00	0.62–1.53	13	1.10	0.59–1.89
MN of kidney (189.0–189.2)	20	0.96	0.58–1.48	13	1.08	0.57–1.84	7	0.79	0.32–1.63	10	0.92	0.44–1.69	10	1.00	0.48–1.84
MN of bladder and other urinary organs (188, 189.3–189.9)	17	1.00	0.58–1.60	12	1.07	0.55–1.88	5	0.85	0.28–2.00	7	0.76	0.31–1.57	10	1.27	0.61–2.34
MN of other and unspecified sites (170–173, 190–199)	125	0.98	0.82–1.17	72	1.17	0.92–1.48	53	0.81	0.61–1.06	63	1.00	0.77–1.28	62	0.97	0.74–1.24
Melanoma (172)	19	1.26	0.76–1.97	14	1.66	0.91–2.79	5	0.75	0.24–1.75	14	1.79	0.98–3.00	5	0.69	0.22–1.61
Brain (191, 192)	23	0.79	0.50–1.19	15	1.04	0.58–1.72	8	0.55	0.23–1.07	11	0.76	0.38–1.36	12	0.82	0.43–1.44
Neoplasms of lymphatic and hematopoietic tissue (200–208)	99	1.05	0.85–1.28	46	1.00	0.73–1.33	53	1.10	0.82–1.44	42	0.90	0.65–1.21	57	1.20	0.91–1.55
Leukemia and aleukemia (204–208)	34	0.96	0.66–1.34	13	0.73	0.39–1.24	21	1.19	0.74–1.82	14	0.79	0.43–1.33	20	1.12	0.68–1.72
NHL (200, 202)	35	0.98	0.68–1.36	16	0.95	0.54–1.54	19	1.01	0.61–1.58	12	0.68	0.35–1.18	23	1.28	0.81–1.92
Myeloma (203)	28	1.85**	1.23–2.67	16	2.31**	1.32–3.76	12	1.46	0.75–2.54	15	2.02*	1.13–3.34	13	1.68	0.89–2.87
Benign and unspecified neoplasms (210–239)	9	0.63	0.29–1.19	2	0.35	0.04–1.25	7	0.82	0.33–1.68	2	0.30	0.04–1.09	7	0.91	0.36–1.87
Diabetes mellitus (250)	72	0.89	0.70–1.12	21	0.64*	0.40–0.98	51	1.06	0.79–1.40	32	0.85	0.58–1.20	40	0.93	0.66–1.26
Blood and blood-forming organ diseases (280–289)	17	1.31	0.76–2.10	10	1.77	0.85–3.26	7	0.95	0.38–1.96	8	1.30	0.56–2.57	9	1.31	0.60–2.50
Mental, psychoneurotic, and personality disorders (290–319)	22	0.64*	0.40–0.96	6	0.34**	0.13–0.75	16	0.94	0.53–1.52	10	0.59	0.28–1.08	12	0.68	0.35–1.19
Multiple sclerosis (340)	6	0.93	0.34–2.03	3	1.46	0.30–4.27	3	0.68	0.14–2.00	2	0.69	0.08–2.49	4	1.13	0.31–2.88
Other nervous system diseases (320–337, 341–389)	61	1.16	0.88–1.48	15	0.64	0.36–1.05	46	1.57**	1.15–2.10	25	0.98	0.64–1.45	36	1.32	0.92–1.82
Heart diseases (390–398, 402, 404, 410–414, 420–429)	1,104	0.93*	0.87–0.98	619	0.92*	0.85–0.99	485	0.94	0.86–1.03	517	0.86**	0.79–0.94	587	1.00	0.92–1.08
Ischemic heart disease (410–414)	861	0.97	0.90–1.03	492	0.93	0.85–1.01	369	1.02	0.92–1.13	399	0.87**	0.79–0.96	462	1.07	0.97–1.17
Hypertension with heart disease (402, 404)	26	0.71	0.47–1.05	12	0.74	0.38–1.30	14	0.69	0.38–1.16	10	0.61	0.29–1.12	16	0.80	0.46–1.30
Other heart diseases (420–423, 425–428, 429.2–429.9)	182	0.86*	0.74–0.99	95	0.89	0.72–1.09	87	0.82	0.66–1.02	92	0.88	0.71–1.08	90	0.84	0.68–1.03
Other circulatory system diseases (401, 403, 405, 415–417, 430–459)	329	1.01	0.90–1.12	141	0.99	0.83–1.16	188	1.02	0.88–1.18	129	0.85	0.71–1.01	200	1.15	0.99–1.32
Respiratory system diseases (460–519)	220	0.83**	0.73–0.95	106	0.80*	0.66–0.97	114	0.87	0.71–1.04	126	0.96	0.80–1.14	94	0.71**	0.57–0.87
Chronic and unspecified bronchitis (490–491)	14	1.99*	1.09–3.35	5	1.33	0.43–3.12	9	2.75**	1.26–5.22	9	2.56*	1.17–4.87	5	1.42	0.46–3.33
Emphysema (492)	22	0.67	0.42–1.01	15	0.78	0.43–1.28	7	0.51	0.20–1.05	14	0.81	0.44–1.36	8	0.51	0.22–1.00
Digestive system diseases (520–579)	122	0.73**	0.60–0.87	57	0.65**	0.49–0.85	65	0.81	0.62–1.03	47	0.56**	0.41–0.75	75	0.89	0.70–1.12
Cirrhosis of the liver (571)	60	0.74*	0.56–0.95	31	0.65*	0.44–0.93	29	0.87	0.58–1.24	25	0.59**	0.38–0.87	35	0.90	0.63–1.25
Genitourinary system diseases (580–629)	39	0.72*	0.51–0.98	12	0.52*	0.27–0.90	27	0.87	0.57–1.27	12	0.48**	0.25–0.84	27	0.92	0.61–1.34
Accidents (E800–E949)	117	0.58**	0.48–0.70	69	0.50**	0.39–0.63	48	0.75	0.55–1.00	69	0.66**	0.51–0.84	48	0.50**	0.37–0.66
Suicide (E950–E978)	40	0.53**	0.38–0.72	29	0.56**	0.38–0.81	11	0.46**	0.23–0.83	25	0.62*	0.40–0.91	15	0.43**	0.24–0.71
Homicide (E960–E978)	9	0.33**	0.15–0.62	6	0.31**	0.11–0.68	3	0.37	0.08–1.07	4	0.28**	0.08–0.72	5	0.38*	0.12–0.90
Other causes (residual codes)	67	0.72**	0.55–0.91	31	0.67*	0.45–0.95	36	0.77	0.54–1.06	23	0.51**	0.32–0.76	44	0.91	0.61–1.22
COD not obtained	143			95			48			72			71		

Abbreviations: COD, cause of death; MN, malignant neoplasm; NA, not applicable; Obs, observed deaths. SMRs are based on U.S. referent rates.

a Deaths and person-years at-risk were accumulated during 1940–1998 for all outcomes except melanoma, NHL, and myeloma, which were accumulated during 1960–1998 due to rate file restrictions.

b Omitted categories include tuberculosis (3 deaths; ICD-9 codes 010–018), MN of other male genital organs (1 death; ICD-9 codes 186–187), skin and subcutaneous tissue diseases (4 deaths; ICD-9 codes 680–709), musculoskeletal system and connective tissue diseases (8 deaths; ICD-9 codes 710–739), and symptoms and ill-defined conditions (10 deaths; ICD-9 codes 780–796, 798, and 799).

* Two-sided *p*-value < 0.05.

** Two-sided *p*-value < 0.01.

**Table 3:** Poisson regression results for cancer outcomes among PCB-exposed workers employed ≥ 90 days.^a^

	No lag			10-Year lag			20-Year lag		
Underlying cause of death,cumulative exposure (unit-years)^b^	Obs	RR	95% CI	Obs	RR	95% CI	Obs	RR	95% CI
All cancers									
< 150	254	1	—	278	1	—	359	1	—
150–< 620	229	1.11	0.93–1.33	232	1.25	1.05–1.50	214	1.31	1.10–1.57
620–< 2,300	238	1.25	1.05–1.50	228	1.31	1.09–1.56	211	1.29	1.08–1.55
≥ 2,300	240	1.28	1.06–1.53	223	1.34	1.11–1.61	177	1.33	1.09–1.61
			*p* = 0.030^c^			*p* = 0.032			*p* = 0.061
Stomach									
< 150	5	1	—	7	1	—	10	1	—
150–< 620	6	1.50	0.46–4.91	5	1.39	0.43–4.52	9	3.50	1.23–9.96
620–< 2300	10	3.16	1.08–9.28	9	3.14	1.13–8.69	6	2.77	0.87–8.76
≥ 2,300	8	2.93	0.93–9.22	8	3.49	1.16–10.52	4	2.38	0.63–9.04
			*p* = 0.12			*p* = 0.043			*p* = 0.62
Stomach (men)									
< 150	3	1	—	4	1	—	7	1	—
150–< 620	5	2.09	0.50–8.76	4	1.97	0.47–8.14	7	4.33	1.26–14.90
620–< 2300	8	4.40	1.16–16.60	8	5.07	1.48–17.34	6	4.53	1.26–16.30
≥ 2,300	8	4.90	1.27–18.90	8	6.30	1.73–22.98	4	3.86	0.89–16.65
			*p* = 0.039			*p* = 0.011			*p* = 0.32
Intestine, excluding rectum									
< 150	21	1	—	23	1	—	31	1	—
150–< 620	26	1.48	0.83–2.62	28	1.80	1.03–3.15	26	1.88	1.08–3.27
620–< 2300	26	1.47	0.82–2.63	26	1.62	0.91–2.89	26	1.74	0.99–3.05
≥ 2,300	27	1.44	0.80–2.59	23	1.41	0.77–2.59	17	1.34	0.70–2.53
			*p* = 0.55			*p* = 0.87			*p* = 0.99
Intestine, excluding rectum (women)									
< 150	7	1	—	9	1	—	13	1	—
150–< 620	15	2.42	0.98–5.93	17	2.76	1.21–6.31	14	2.54	1.11–5.80
620–< 2300	19	2.65	1.10–6.39	16	2.19	0.94–5.12	20	3.04	1.38–6.71
≥ 2,300	20	2.82	1.16–6.86	19	2.75	1.18–6.42	14	2.63	1.11–6.24
			*p* = 0.16			*p* = 0.18			*p* = 0.26
Rectum									
< 150	5	1	—	5	1	—	7	1	—
150–< 620	5	1.13	0.33–3.93	5	1.24	0.36–4.30	3	0.76	0.20–2.98
620–< 2300	1	0.20	0.02–1.77	1	0.22	0.03–1.88	3	0.62	0.16–2.44
≥ 2,300	8	1.35	0.43–4.26	8	1.56	0.50–4.92	6	1.60	0.51–4.97
			*p* = 0.36			*p* = 0.27			*p* = 0.26
Biliary passages, liver, and gallbladder									
< 150	2	1	—	4	1	—	6	1	—
150–< 620	3	1.72	0.29–10.33	2	0.75	0.14–4.20	1	0.44	0.05–3.89
620–< 2300	6	3.07	0.61–15.43	6	1.93	0.52–7.15	6	2.28	0.64–8.11
≥ 2,300	9	4.16	0.87–19.80	8	2.58	0.72–9.22	7	3.23	0.90–11.59
			*p* = 0.071			*p* = 0.094			*p* = 0.039
Breast									
< 150	29	1	—	34	1	—	45	1	—
150–< 620	26	1.09	0.64–1.85	26	1.21	0.72–2.04	23	1.20	0.70–2.04
620–< 2300	19	0.79	0.44–1.43	15	0.68	0.36–1.27	15	0.75	0.41–1.40
≥ 2,300	27	1.32	0.75–2.30	26	1.44	0.82–2.52	18	1.25	0.68–2.30
			*p* = 0.26			*p* = 0.17			*p* = 0.52
Other/unspecified parts of uterus
< 150	1	1	—	1	1	—	2	1	—
150–< 620	2	2.46	0.22–27.3	2	2.93	0.26–32.9	2	2.33	0.30–18.2
620–< 2,300	5	6.45	0.72–57.4	6	9.17	1.03–81.6	6	6.80	1.13–40.8
≥ 2,300	6	8.77	0.96–80.3	5	8.73	0.90–85.0	4	5.58	0.80–39.1
			*p* = 0.058			*p* = 0.14			*p* = 0.23
Ovary, fallopian tube, and broad ligament
< 150	6	1	—	6	1	—	9	1	—
150–< 620	7	1.53	0.51–4.56	8	2.27	0.77–6.66	6	1.77	0.59–5.33
620–< 2,300	8	2.12	0.72–6.26	7	2.39	0.77–7.41	7	2.53	0.85–7.48
≥ 2,300	7	2.67	0.83–8.61	7	3.61	1.10–11.86	6	3.63	1.11–11.93
			*p* = 0.17			*p* = 0.10			*p* = 0.073
Prostate
< 150	4	1	—	4	1	—	6	1	—
150–< 620	5	1.51	0.40–5.61	5	2.09	0.55–7.91	4	2.15	0.57–8.10
620–< 2,300	7	2.82	0.82–9.63	7	3.90	1.13–13.47	8	5.19	1.69–15.94
≥ 2,300	18	6.05	2.01–18.18	18	8.49	2.72–26.51	16	10.33	3.49–30.57
			*p* = 0.0001			p = < 0.0001			*p* = < 0.0001
Melanoma^d^
< 150	9	1	—	9	1	—	12	1	—
150–< 620	2	0.27	0.06–1.27	2	0.36	0.08–1.69	2	0.46	0.10–2.11
≥ 620	6	0.65	0.23–1.85	6	0.85	0.29–2.48	3	0.50	0.13–1.92
			*P* = 0.83			*p* = 0.88			*p* = 0.40
Brain
< 150	10	1	—	11	1	—	13	1	—
150–< 620	5	0.62	0.21–1.82	4	0.58	0.18–1.85	5	0.99	0.34–2.92
620–< 2,300	3	0.44	0.12–1.60	4	0.67	0.21–2.15	1	0.21	0.03–1.63
≥ 2,300	3	0.46	0.12–1.73	2	0.36	0.08–1.70	2	0.52	0.11–2.48
			*p* = 0.32			*p* = 0.22			*p* = 0.33
NHL
< 150	10	1	—	11	1	—	12	1	—
150–< 620	13	1.57	0.69–3.59	13	1.70	0.75–3.83	12	2.27	0.98–5.30
620–< 2,300	3	0.46	0.12–1.67	2	0.32	0.07–1.48	4	0.89	0.27–2.89
≥ 2,300	7	1.18	0.43–3.26	7	1.26	0.46–3.48	5	1.30	0.41–4.11
			*p* = 0.99			*p* = 0.91			*p* = 0.89
Myeloma
<150	5	1	—	6	1	—	9	1	—
150–< 620	6	1.48	0.45–4.85	6	1.42	0.46–4.44	4	0.89	0.27–2.98
620–< 2,300	9	2.44	0.81–7.33	8	2.03	0.69–5.96	8	1.79	0.66–4.87
≥ 2,300	8	1.90	0.61–5.94	8	1.83	0.61–5.50	7	1.59	0.55–4.63
			*p* = 0.48			*p* = 0.44			*p* = 0.40

Obs, observed deaths. Poisson regression analysis (log-linear model) was performed using the GENMOD procedure in SAS. RRs were adjusted for sex (reference = male, female), age (reference = < 50, 50–59, 60–69, ≥ 70 years), and calendar year (reference = < 1970reference = < 1970–79, 1980–89, ≥ 1990).

a Deaths and person-years at-risk were accumulated during 1940–1998 for all outcomes except melanoma, NHL, and myeloma, which were accumulated during 1960–1998; workers with time in an unknown job code (*n* = 486) were excluded.

b Cumulative exposure was estimated using the combined inhalation–dermal job exposure matrix, which weights inhalation and dermal exposure equally.

c Trend test *p*-value.

d The two highest exposure categories were combined for melanoma because there were no observed deaths in the highest exposure category.
